# Simulation of a flash-flood event over the Adriatic Sea with a high-resolution atmosphere–ocean–wave coupled system

**DOI:** 10.1038/s41598-021-88476-1

**Published:** 2021-04-30

**Authors:** Antonio Ricchi, Davide Bonaldo, Guido Cioni, Sandro Carniel, Mario Marcello Miglietta

**Affiliations:** 1 Department of Physical and Chemical Sciences, University of L’Aquila, 67100, Via Vetoio (Coppito 1, Edificio “Renato Ricamo”), L’Aquila, Italy; 2 Center of Excellence in Telesensing of Environment and Model Prediction of Severe Events (CETEMPS), Via Vetoio (Coppito 1, Edificio “Renato Ricamo”), L’Aquila, 67100 Italy; 3 CNR-ISMAR, Institute of Marine Sciences, Arsenale Tesa 104, Castello 2737/F, 30122 Venice, Italy; 4 CNR-ISAC, Padua/Lecce, Italy; 5 STO CMRE, V.le San Bartolomeo 400, I-19126, La Spezia, Italy; 6 Max Planck Institute for Meteorology, Hamburg, Germany

**Keywords:** Atmospheric dynamics, Environmental sciences, Natural hazards, Physical oceanography

## Abstract

On the morning of September 26, 2007, a heavy precipitation event (HPE) affected the Venice lagoon and the neighbouring coastal zone of the Adriatic Sea, with 6-h accumulated rainfall summing up to about 360 mm in the area between the Venetian mainland, Padua and Chioggia. The event was triggered and maintained by the uplift over a convergence line between northeasterly flow from the Alps and southeasterly winds from the Adriatic Sea. Hindcast modelling experiments, using standalone atmospheric models, failed to capture the spatial distribution, maximum intensity and timing of the HPE. Here we analyze the event by means of an atmosphere-wave-ocean coupled numerical approach. The combined use of convection permitting models with grid spacing of 1 km, high-resolution sea surface temperature (SST) fields, and the consistent treatment of marine boundary layer fluxes in all the numerical model components are crucial to provide a realistic simulation of the event. Inaccurate representations of the SST affect the wind magnitude and, through this, the intensity, location and time evolution of the convergence zone, thus affecting the HPE prediction.

## Introduction

Recent studies^1,2^ have shown that Heavy Precipitation Events (HPEs) in the Mediterranean coastal regions are in many ways dependent on Sea Surface Temperature (SST). SST magnitude and spatial patterns control the air-sea energy exchange, thus modifying the environment where precipitating systems develop by modulating evaporation^3^, modifying the atmospheric low-level stability^4^, and also changing the amount of precipitable water through moistening of the marine boundary layer (MABL). In this way, the air-sea interaction affects the structure and organization of precipitating systems, their lifecycle, severity, propagation speed and track, thus impacting the rain intensity^1,5,6^. Also, they may induce a rapid intensification of deep moist convection, leading in the Mediterranean to extreme precipitation amounts^7,8^, to the transition of baroclinic cyclones into cyclones with tropical characteristics^9,10^, or to the intensification of mesocyclonic waterspouts which, after moving inland, may produce devastating consequences^11^.

In search of an accurate representation of HPEs in NWP (Numerical Weather Prediction) models, increasing attention is now being paid to coupled atmosphere–ocean–wave numerical systems, since they treat in a consistent way the air-sea interface processes relative to heat, mass and momentum exchanges^12^. However, the difficulties related to their implementation (need of extensive computational resources) and validation (lack of data over the sea) still prevent these tools from being routinely employed for operational purposes.

The aim of this study is to explore how the use of a coupled modelling system may influence the simulation of regional and local scale atmospheric patterns of a HPE over the Mediterranean region and to investigate whether it may provide more predictive skills than standalone atmospheric models. The event investigated in this study took place in the Venice lagoon on September 26, 2007, locally producing more than 360 mm in 12 h^13,14^. This event caused severe damages to infrastructures, public services and private property, and was poorly predicted by both real-time operational models and hindcast simulations. Hereafter, we investigate the relevance of the effect of small-scale SST features, which are not resolved in the lower boundary conditions generally used to drive NWP models, for the proper simulation of rainfall amount and localization in this HPE. By doing this, an outline of the processes controlling these events will emerge, alongside with a discussion on the implications and requirements in terms of model coupling and parameterization strategies.

“Case study and simulation setup” section describes the case study and the numerical setup of the simulations implemented here. Results are shown in “Results” section, followed by discussion in “Discussion” section. Conclusions are finally drawn in “Conclusions” section.

## Case study and simulation setup

**Figure 1:**
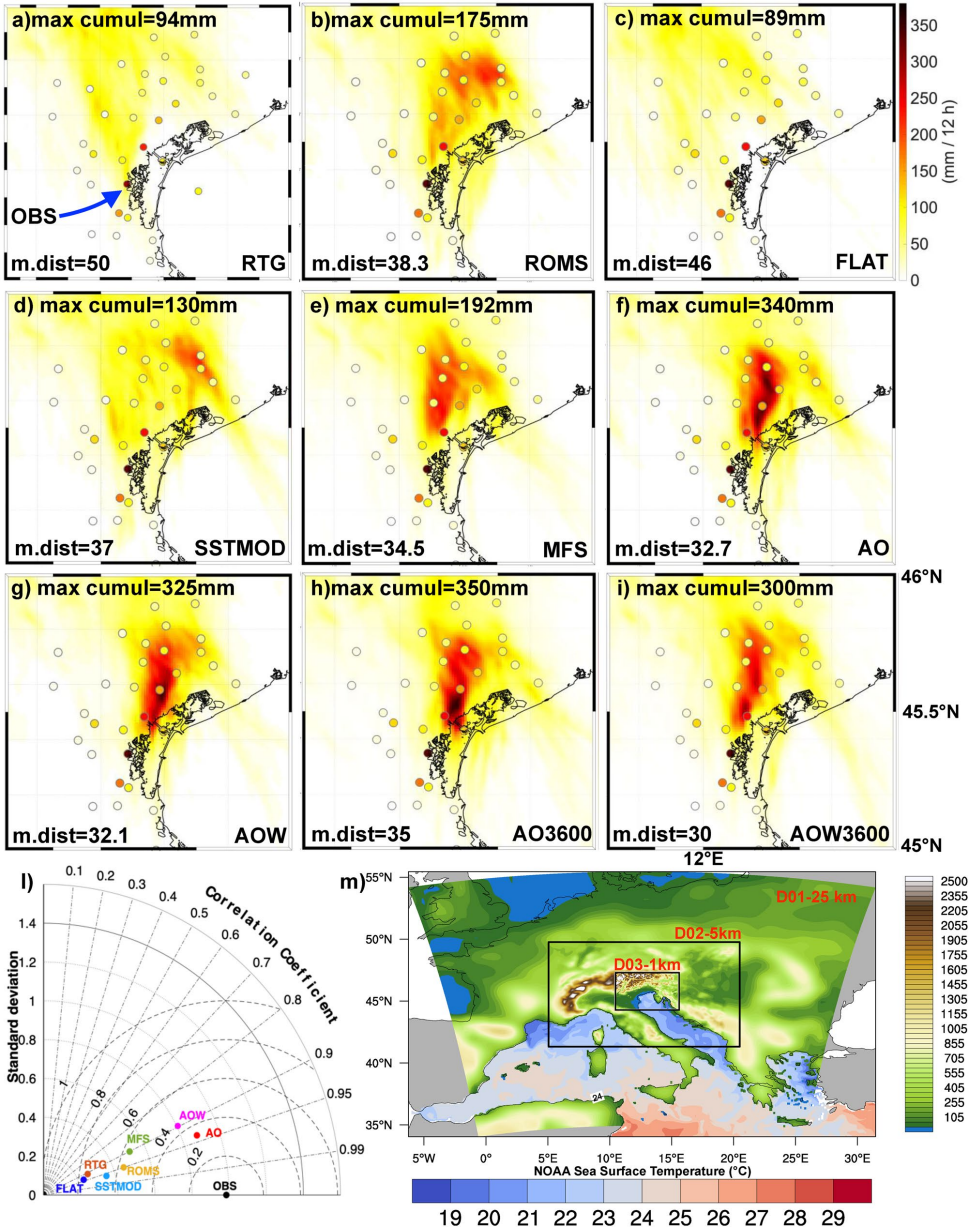
Panels (**a**–**i**) 6 h accumulated precipitation observed in each ARPAV station (coloured dots), together with simulated precipitation (mm) as resulting from the different runs. Plots refer to the period 06–12 UTC, 2007 September, 26: *max cumul* is the sum of maximum hourly values associated with the storm cell; *m.dist* is the average distance (in km) between the storm location and Campagna Lupia (where the most intense rainfall was observed and highlighted with “OBS” in panel (**a**); panel l: Taylor diagram of the timeseries of hourly rainfall maximum associated with the storm cell. Panel m shows the numerical domains, topography and satellite SST (resolution 8.3 km) at 06 UTC.

The HPE near Venice of September 26, 2007^15^ was generated by a Mesoscale Convective System (MCS). The latter was triggered and sustained by the convergence between dry and cold air coming from the Alps (barrier wind), with warmer and humid air from the southern Adriatic (Sirocco wind). These two different air masses met in proximity of the northeastern Italian coastline, triggering intense convective activity. Deep moist convection remained active in the same area for about 6 h (from 04 to 10 UTC) and was organized in the V-shape typical of a back-building convective system^16^, with an estimated cloud top temperature of about − 55 °C at 12 km height^15^. The multicell system remained quasi-stationary, slowly moving towards the sea^14^, and caused intense precipitation in an area of about 10 km^2^, with accumulated rainfall of 360 mm in 6 h (Fig. 1) in the small town of Campagna Lupia, about 180 mm in Mira and 130 mm in Venice. The spatial pattern and the intensity of the rainfall were retrieved from observational records provided by the Veneto Region Environmental Protection Agency (*ARPAV*) rain gauge network^15^. The three-dimensional features of the system were reconstructed based on the data from Teolo C-Band radar located in Monte Grande (472 m a.s.l., approximately 40 km south-west of the event), processed by ARPAV. This event is classified in the category of “Upstream” HPEs^15^, where low-level blocking conditions persist, the upstream profile is unstable and the level of free convection is located at low altitude, thus the uplift over the barrier wind cold layer is strong enough to trigger convection. In order to simulate the HPE, we employ the COAWST numerical framework (Coupled Ocean Atmosphere Waves Sediment Transport model^17–19^), which consists of a coupled system among the atmospheric model WRF^20^ (Weather Research and Forecasting system), the oceanic model ROMS^21^ (Regional Oceanographic Modelling System), and the wave model SWAN^22^ (Simulating WAves in Nearshore). WRF is a state-of-the-art numerical weather prediction system that solves the fully compressible, nonhydrostatic Euler equations. In the present study, the version ARW-3.8 has been configured in a 3-domain setup with 1:5 nesting ratio, from 25 km down to 1 km grid spacing (the coarser grid covers the whole central Europe, the inner domain the northern Adriatic Sea). As a first step, in order to identify the best numerical and physical configuration, several stand-alone sensitivity tests were carried out. In detail, we have tested the sensitivity to the number of vertical levels (from 45 to 75, in step of 10), to the soil dataset (USGS and MODIS), and, following the results in^1^, to different parameterization schemes available in WRF, relative to microphysics, planetary boundary layer, cumulus convection (in the coarser domains), and nesting technique (2-way vs 1-way). The most accurate results, in terms of accumulated precipitations and localization of HPE, were obtained using 55 vertical levels (with the first level at 15 m above the ground), the MODIS soil dataset (ORNL DAAC. 2018. MODIS and VIIRS Land Products Global Subsetting and Visualization Tool), initial and boundary conditions provided by the FNL dataset (Final Reanalysis of the Global Forecasting System), and 2-way nesting technique. The sensitivity to physics showed that better results were obtained using the Kain-Fritsch cumulus scheme^23^ only for the two coarser domains, the WSM 5-class microphysics scheme^24^, the Mellor-Yamada-Janjic Planetary Boundary Layer scheme^25^, and RRTMG^26^ (Rapid Radiative Transfer Model Radiation Scheme) for longwave and shortwave radiation. ROMS and SWAN use the same 1-km grid covering the entire Adriatic Sea, with an open boundary at the Otranto Strait. ROMS entails the 3-D hydrostatic formulation of the Reynolds-averaged Navier–Stokes equations, discretizing the water column into 30 terrain-following vertical levels, whereas SWAN provides a phase-averaged description of the generation, propagation and dissipation of the sea state by dividing the spectral domain into 25 logarithmically-spaced frequencies and 36 directions. This configuration was successfully used in previous works in the Adriatic Sea^27,28^, to which the reader is referred for further details. The coupling frequency, which plays a fundamental role in the description of the energy fluxes at the air-sea interface, was set to 600 s for the reference run, since this value provided better results in terms of statistical indices compared to a sensitivity test using 3600 s, as shown in Fig. 1.

**Figure 2:**
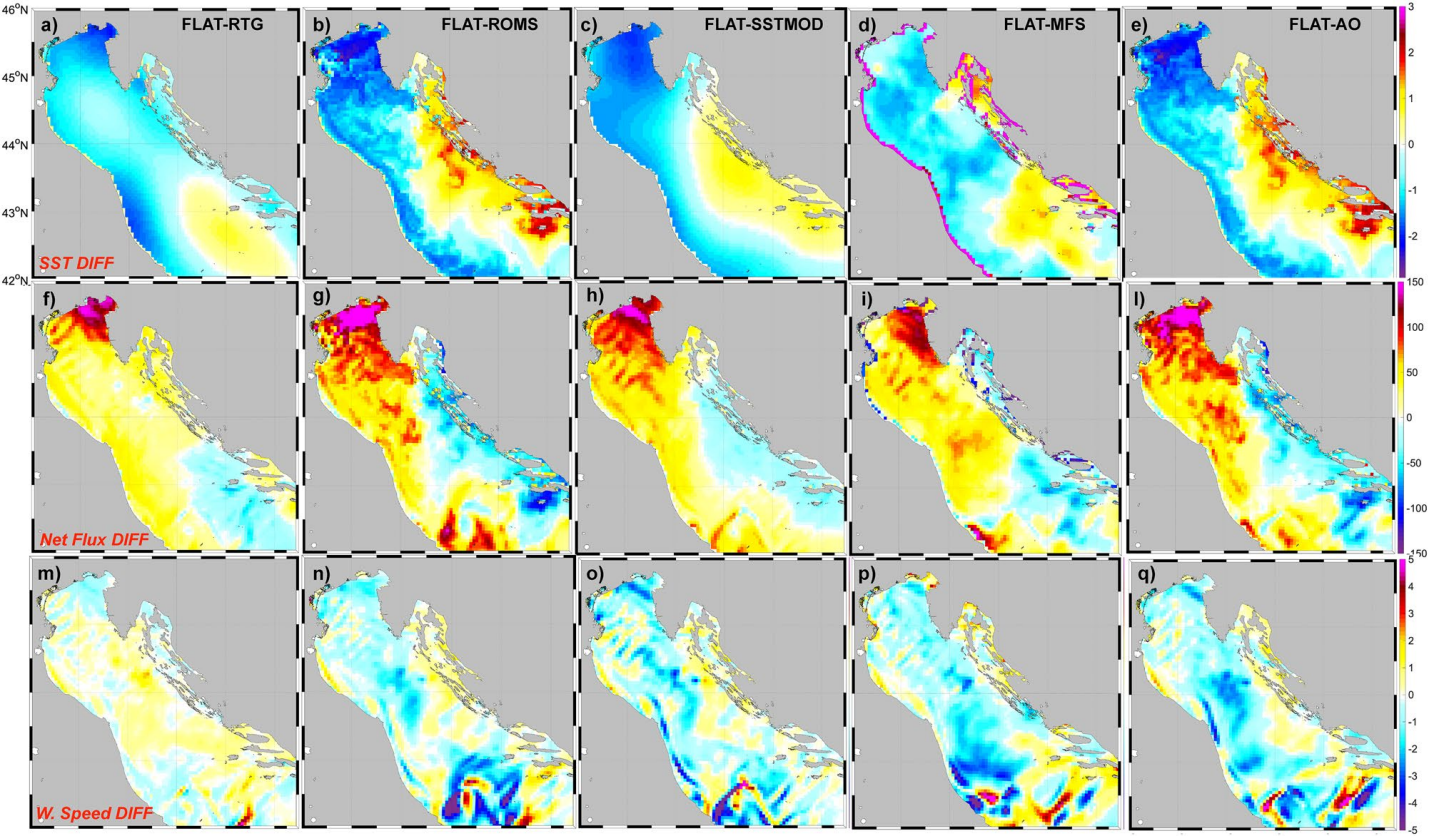
Upper row: Sea Surface Temperature (SST) difference between the FLAT run and the other simulations (in °C) at 06 UTC, September 26. Middle row: Surface Heat Fluxes difference between the FLAT run and the other simulations (in W m^−2^). Bottom row: 10 m wind speed difference between the FLAT run and the other simulations over sea surface. Due to the marked similarity in terms of SST with the AO run, the results for AOW run are not shown here (see Fig. 3).

The COAWST modeling suite has been tested in 2 different groups of configurations (shown in Table 1), all starting at 00 UTC, September 25 and ending at 00 UTC, September 27. In the first group, COAWST has been used as a stand-alone atmospheric system (the WRF model). The simulations differ in terms of SST forcing and are as follows:

1. *RTG:* initial and boundary conditions are taken from satellite data in the NOAA database (RTG_SST), provided at 8.3 km resolution every 6 h (Fig. 2a);
2. *ROMS:* SST fields are extracted from the spin-up simulation^2^ (a one-way coupled atmosphere–ocean simulation starting at 00 UTC, September 1, with ocean variables initialized by the CMEMS fields^29^) used to initialize the AOW and AO run. Boundary conditions are provided every 6 h to force WRF as a standalone model. Thus, compared to the RTG simulation, this run uses a higher-resolution dataset, obtained from a coupled model simulation; compared to the coupled run, it has the same initial condition but it does not treat in a consistent way the air-sea fluxes;
3. *SSTMOD:* SST is the same as that used in run ROMS, but at 8.3 km grid spacing. Thus, this run is intermediate between runs ROMS and RTG and its purpose is to evaluate the effect of resolution (in comparison with ROMS) and of the data quality (in comparison with RTG);
4. *FLAT:* SST is uniform across the basin. The SST average value is calculated starting from run ROMS and applied to the entire grid. It is designed to evaluate the influence of SST gradients and patterns on atmospheric dynamics;
5. *MFS:* the SST is obtained from the CMEMS-MFS dataset^29^ at 4.5 km grid spacing, updated every 6 h. Consequently the run MFS is similar to ROMS, but starts from a different modelling dataset, thus it is used to estimate the impact of a different modelling dataset in comparison with the run ROMS.
  In the second group, COAWST was used as a coupled numerical system. The initial conditions are the result of the spin-up simulation.
6. *AO:* WRF and ROMS are 2-way coupled;
7. *AOW:* this is a fully coupled atmosphere–ocean–wave setup, envisaging the 2-way feedback of both atmosphere and ocean models with the wave model (SWAN), as described in^19,28^;
8. *AO3600:* this coupled run is identical to run AO, except for the coupling interval being 3600 s instead of 600 s;
9. *AOW3600:* same as in AOW, but with 3600 s as coupling interval.

**Table 1:** The table summarizes the types of runs proposed and the approaches followed in terms of type of Sea Surface Temperature, resolution of the SST data, updating of the SST data and coupling.

RUNS TYPE	RTG	ROMS	SSTMOD	FLAT	MFS	AO	AOW	AO3600	AOW3600
SST type	Satellite RTG_SST	SpinUp	ROMS degraded	Homogeneous (Mean Basin from ROMS run)	Operational Model	Coupling WRF + ROMS	Coupling WRF + ROMS + SWAN	Coupling WRF + ROMS	Coupling WRF + ROMS + SWAN
SST source data resolution	8.3 km	1 km	8.3 km	/	4.5 km	1 km	1 km	1 km	1 km
SST upgrade	6 h	6 h	6 h	6 h	6 h	600 s	600 s	3600 s	3600 s
Coupling	No	No	No	No	No	Yes	Yes	Yes	Yes
Coupling frequency	/	/	/	/	/	600 s	600 s	3600 s	3600 s

## Results

In order to compare the results from the different model setups, we first focus on the modelled SST field. The first row in Fig. 2, which shows the differences in SST between the FLAT run and each model simulation at 06 UTC, September 26 (shortly before the HPE) highlights significant differences in the northern and central Adriatic Sea. The SST data obtained from satellite (RTG; Fig. 2a) is about 1.5 °C colder than the 1-km field (ROMS; Fig. 2b) over the northern Adriatic Sea and along the Italian coast, while it is 1.5–2 °C warmer near the Croatian coast. In semi-enclosed basins, particularly in rough coastal areas, satellites may be significantly affected by scattering effects due to land and river plume contamination^28^, causing SST bias between 1 and 4 °C^8,19^.

**Figure 3:**
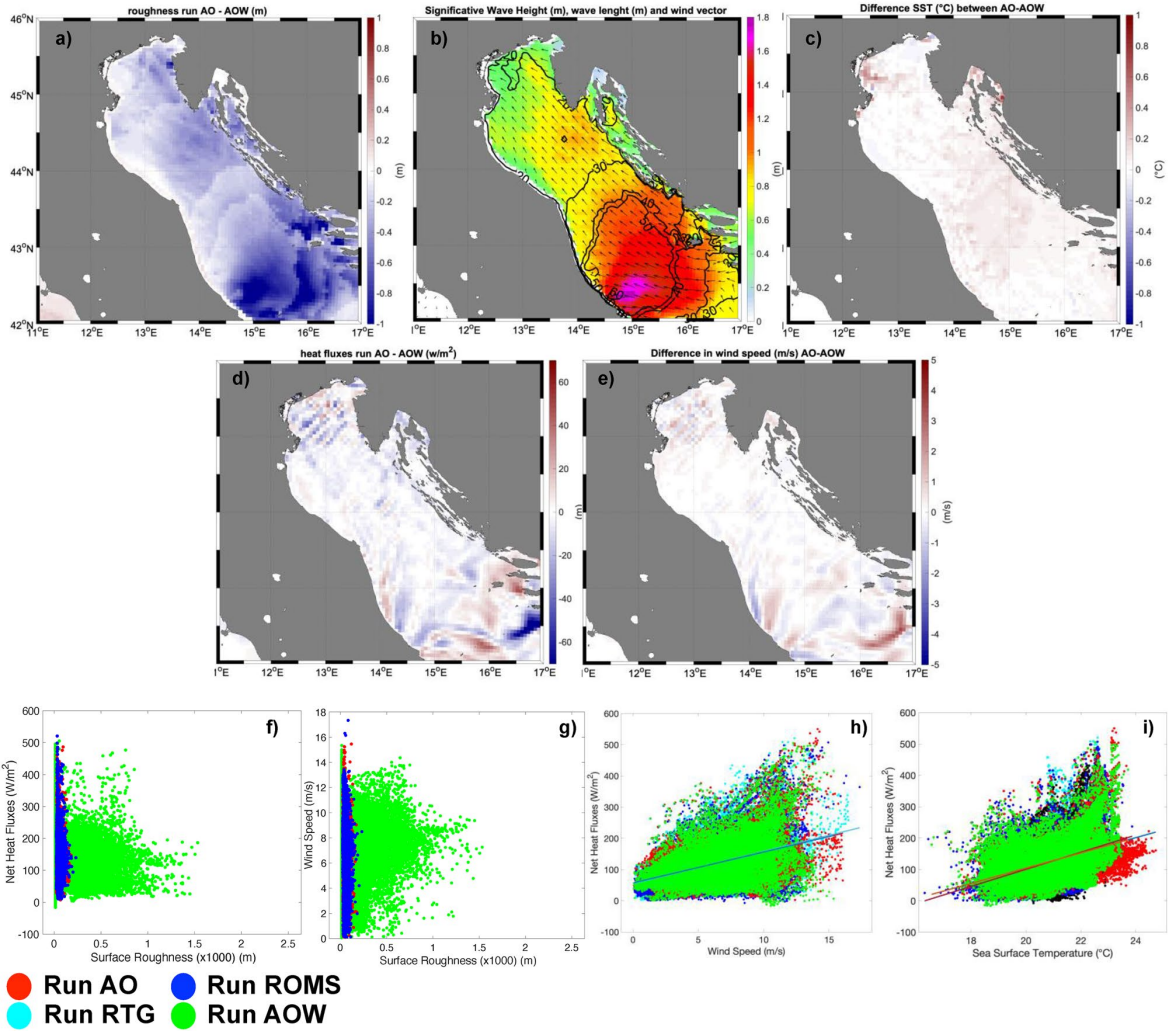
(**a**) Difference in surface roughness, (**c**) in SST, (**d**) in surface heat fluxes, (**e**) in 10 m wind speed between run AO and run AOW, (**b**) significant height of the wave (shaded) and the peak length of the wave fields (contours) in AOW run, (**f**) correlation between heat fluxes and surface roughness, (**g**) between heat fluxes and surface roughness, (**h**) between heat fluxes and 10 m wind, (**i**) between heat fluxes and SST.

The SST pattern in SSTMOD is similar to that of ROMS, but it misses the small-scale features, as a consequence of the coarser resolution. Although the MFS and the ROMS run are both modelling outputs, significant differences can be identified. These are not only due to the different resolution (4.5 km vs 1 km), but also to differences in model formulation and input (e.g., freshwater sources) description. The differences are remarkable in the area north-east of Ancona, where the two fields show respectively a cold and a warm plume extending from the Croatian coast, with differences up to 3 °C. The differences among the coupled runs (Fig. 3c) are more limited, within a few tenths of 1 °C, mainly concentrated in the North Adriatic.

Comparing ROMS with the coupled approaches (Fig. 2b vs Fig. 2e), we note that the coupling induces a different representation of the Po river plume, which is advected northward, thus leading to more mixed and milder waters upstream of the convergence line (pink area in Fig. 2e). These differences can be partly attributed to the different intervals of data communication (6 h in ROMS, 10 min in AO/AOW). Also, as shown in Fig. 3c, the coupling with the waves in AOW induces a cooler SST, as a consequence of the stronger mixing^28^ of the river water mass compared to AO. The areas with warmer SST induce greater (negative) surface heat fluxes (Fig. 2, second row). As a consequence, in the northern Adriatic, in the area where the convergence line develops, the sensible heat fluxes in ROMS and in the coupled runs can be up to 150 W m^−2^ higher than in the RTG and MFS simulations, and 100 W m^−2^ than in the SSTMOD case. The warm temperature in the sub-basin in the ROMS and AO fields is mainly associated with the Po river plume, which is hardly identified in satellite (RTG) and low-resolution modelling data (MFS). In the northern Adriatic and in the central part of the basin, not only the satellite SST (run RTG) is smaller than that in the coupled simulations (Fig. 2a vs Fig. 2e), but it is also associated with weaker winds (Fig. 2m vs Fig. 2q), up to 2.5 m/s. Such a difference in winds cannot be attributed to differences in pressure patterns (Fig. SUPPL.2), increasing only after the frontal passage, but it is mainly due to the different transfer of energy from the sea to the atmosphere, caused by the different SST values and patterns. This is typical in areas of long fetch, after the wind has crossed hundreds of km in the same direction^30–36^, such as the northern Adriatic in this case study. Therefore, the wind intensity is modulated by the distribution of the SST field, which influences the extraction of energy from the sea. Comparing the different coupled model configurations, non-negligible differences are evident. Technically, the difference between the AO and AOW runs lies in the addition of the wave model: waves transfer energy along the water column, increase the water mixing^37–39^ and affect, through the wave roughness, the winds in the lowest meters of the atmosphere^40,41^, thus the drag coefficient and the heat fluxes from the sea surface. In the AO simulation, the WRF model imposes a roughness calculated with Charnock^42^ scheme. In the AOW run the roughness is based on Oost^43^ parameterization (here preferred to other schemes available in COAWST), where the roughness depends on the wave age (1996 ASGAMAGE experiment). Figure 3a shows that the AOW run generates greater roughness, in particular in areas with greater significant height (Fig. 3b), e.g. the central Adriatic where waves are greater than 1.6 m and the peak lengths about 50 m. In the other areas of the basin, roughness is only slightly greater in AOW runs. Figure 3c shows that the SST of the AO run is slightly warmer than that of the AOW run, in particular in the central Adriatic and east of Venice, north of the Po river plume. The latter differences can be considered as a cumulative effect of the water mixing associated with the waves in the downstream end of the basin in the AOW run, which cools down the Po river plume as it is advected northward. Furthermore, the AO run produces, especially in the North Adriatic, slightly higher wind speed U (about 0.1 m/ higher near the convergence line and 0.8 m/s east of Venice; Fig. 3e and Table 1), due to the smaller roughness over the sea surface. However, despite the water cooling and the overall lower wind speed (Fig. 3e), the AOW run shows more intense surface heat fluxes (Fig. 3d) in the North Adriatic, due to the increase of both the roughness and drag coefficient. Figure 3f–i show that, differently from the other runs, in the AOW run the surface roughness is a limiting factor for high wind intensity and SST, but not for the heat fluxes. In contrast, the AO run produces higher SST values (Fig. 3i) and, due to the absence of the wave-induced mixing, heat fluxes are mainly driven by the thermal forcing, while they are mainly guided by surface roughness in AOW.

**Figure 4:**
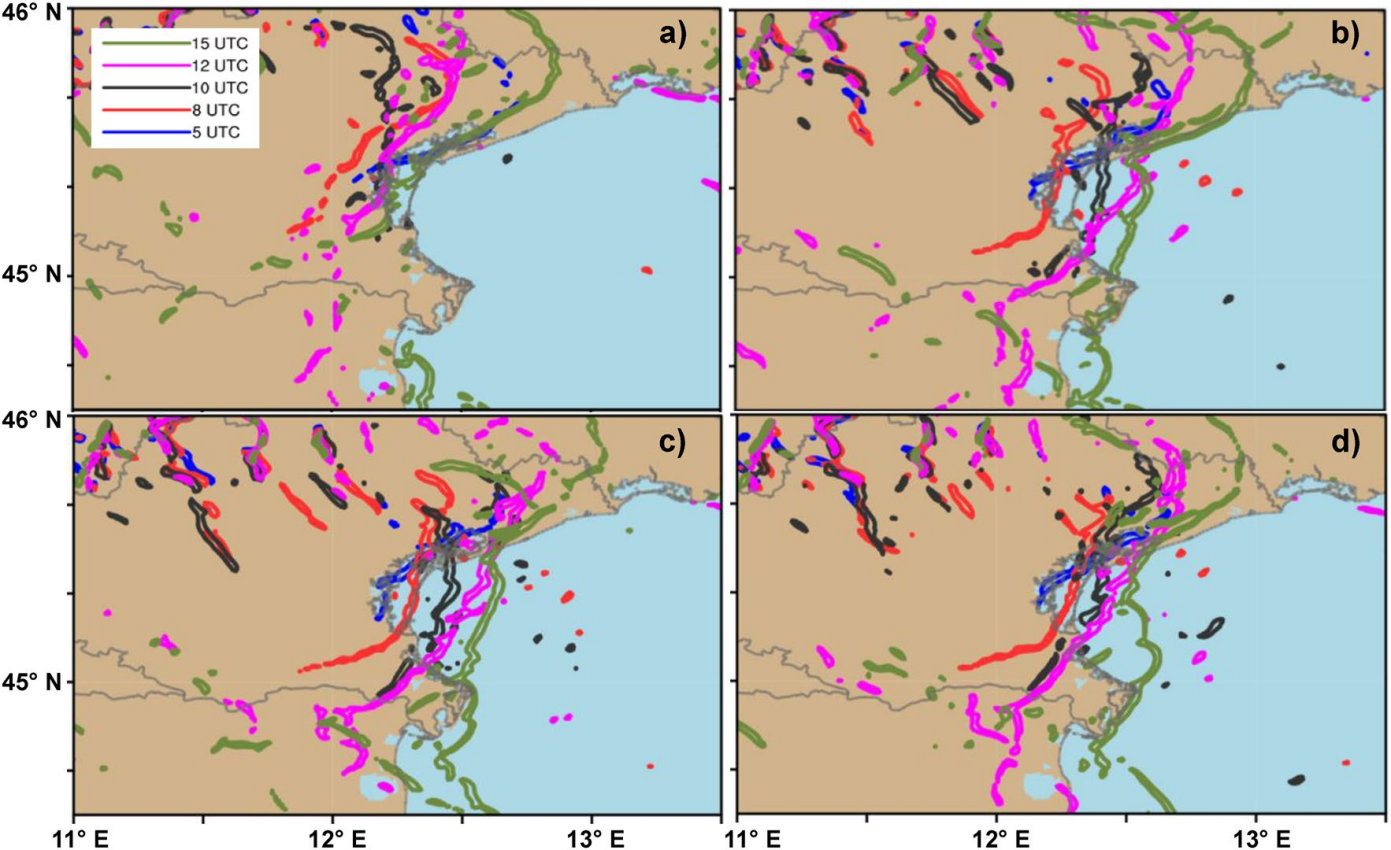
Convergence line (0.003 s^−1^ isoline) location at 05, 08, 10, 12, 15 UTC on September 26, for run RTG (**a**), ROMS (**b**), AO (**c**), and AOW (**d**).

**Figure 5:**
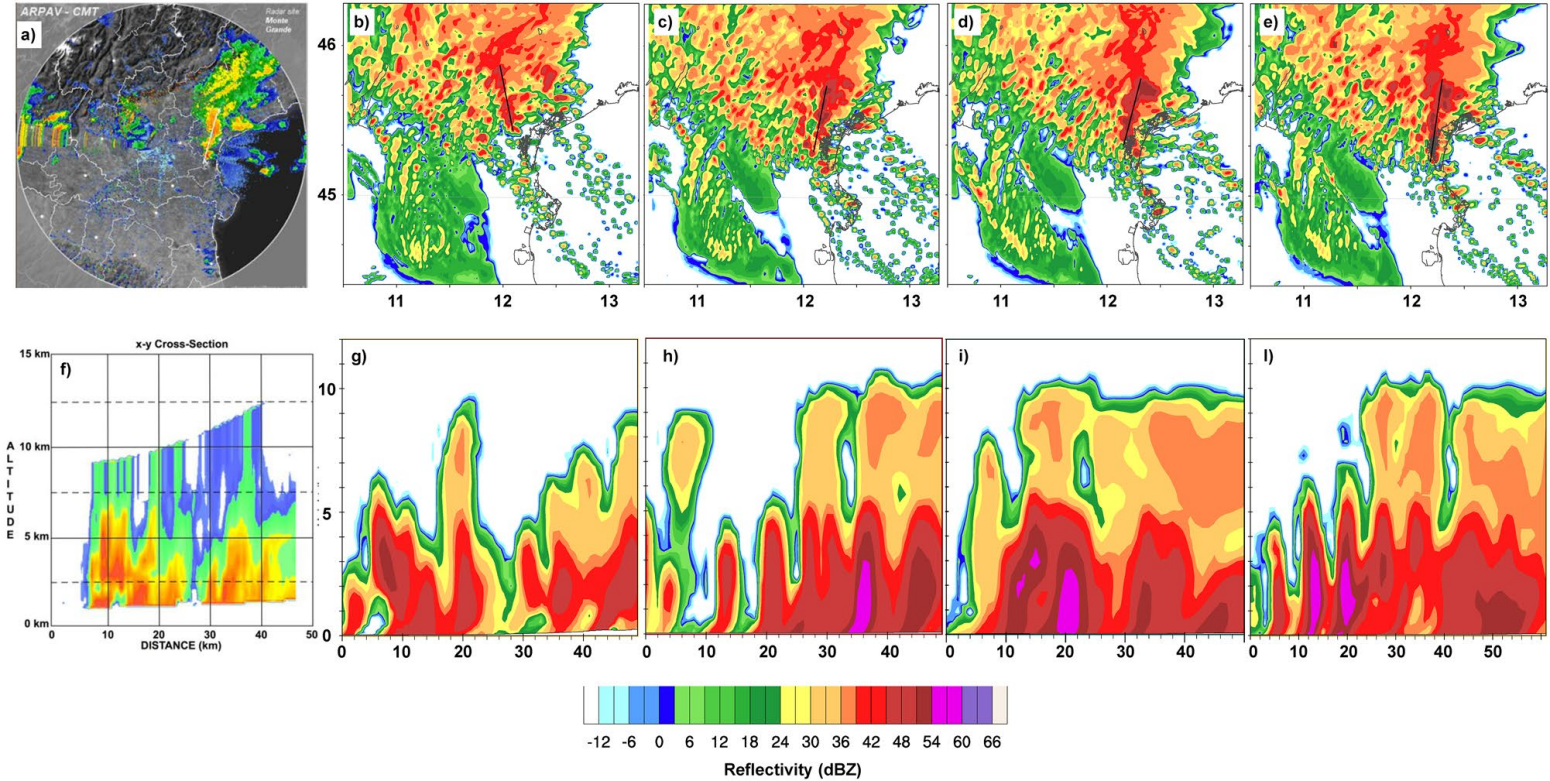
Upper row: (**a**) 2-D radar reflectivity data (Vertical Maximum Intensity) in the HPE region; synthetic radar data simulated in (**b**) RTG, (**c**) ROMS, (**d**) AO and (**e**) AOW; (**f**) cross section of the storm radar reflectivity acquired by the radar in Teolo at 06 UTC, 26 September 2007; (**g**)–(**l**) reflectivity vertical cross sections along the axes of most intense convection (whose locations are in panels (**b**)–(**e**) for (**g**) RTG, (**h**) ROMS, (**i**) AO and (**l**) AOW.

As expected, the aforementioned differences in air-sea interactions significantly affect the precipitation distribution. Figure 1 shows the precipitation amounts resulting from the different model configurations compared with the data from the meteorological stations of the ARPAV network. The analysis of the precipitation simulated in the different runs is based on the maximum 6-h accumulated precipitation (6–12 UTC) and on the average distance (in km) between the simulated and the observed hourly maximum. Figure 1 shows that a more accurate representation of the air-sea interaction processes leads to a more realistic rainfall amount and location. The rainfall amount ranges from 89 mm in run FLAT to more than 300 mm in the coupled runs, and the average distance between the simulated and the observed storm ranges from 50 km (RTG) to about 30 km in the coupled runs. Figure SUPPL3 clearly shows that the coupled runs outperform the standalone runs in terms of precipitation amount and distribution. As discussed before, the different distribution of SST controls the energy fluxes between the ocean and the atmosphere, affecting the wind fields. One might mistakenly think that this only happens on the sea surface, but, in reality, there are differences simulated on a large scale and on the entire computing domain. This leads us to conclude that there is a direct (thermal) component induced by the local interaction between sea and atmosphere and an indirect one due to the different distribution of heat as the simulation progresses, affecting a wider area. Both AO and AOW coupled runs are able to simulate correctly the total amount of precipitation (with a maximum value of about 340 mm and 325 mm respectively, while 360 mm have been recorded in the station of Campagna Lupia). The coupled runs are the only ones able to reproduce the rainfall along a stationary line extending from the Venetian lagoon northward. The location of the rainfall maximum in AO and AOW runs is slightly shifted to the north-east of Campagna Lupia. By increasing the coupling interval between models, similar results are observed in runs AO3600 and AOW3600 in terms of rainfall amount and distance between the observed and the simulated cell (35 and 30 km). Figure 1e shows the Taylor diagram for all the simulations, considering the time series of the maximum hourly accumulated rainfall (thus following the cell evolution). Results show that the correlation (between 0.89 and 0.93) is high for all experiments, but the standard deviation is much closer to the observations in the AO and AOW runs. The movement of the convergence line obtained by different numerical runs is depicted in Fig. 4. This feature provides a dynamical perspective on the difference in the spatial distribution of precipitation, which will be further discussed in “Discussion” section. The structure of the storm is shown in Fig. 5, where modelled reflectivity fields are compared against radar observations. Figure 5c–e and h–l show that the precipitation simulated in ROMS, AO and AOW runs develop from multiple cells arranged along the convergence line (north–south), in good agreement with the reflectivity measured by ARPAV (Fig. 5a,f), while the RTG run (Figs. 4g and 5b) develops cells with lower reflectivity values and shallower vertical development.

When we explore the reflectivity within the HPE region (Fig. 5a), the ROMS run (Fig. 5c) reproduces a storm less intense compared to the coupled runs AO and AOW (Fig. 5d,e). In particular, the coupled runs are able to reproduce the V-shaped structure of the storm at the right location with the proper intensity (Fig. 5e). Also, in terms of reflectivity field, the AO and AOW runs show values and spatial patterns comparable with the radar data. Satellite observations highlight the presence of possible overshooting tops^15^, which are consistent with the high cloud tops (at about 11 km) simulated in the ROMS and coupled runs (Fig. 5h–l).

## Discussion

Operational NWP models were not able to properly simulate the rainfall amount and location of the analyzed HPE, therefore missing their function of alerting the local authorities about the exceptional amount of rain to be expected. The analysis of different Limited Area Model (LAM) simulations^15^ highlighted a large variability depending on the large scale forcing, on the model formulation, and on the time of initialization. Our results show that the explicit and consistent description of the air-sea interactions leads to a noticeable improvement in the model skills. However, in order to clearly understand why this result is achieved, we need to summarise the state of the atmospheric system with a limited number of bulk parameters. To this aim, the quantities describing the average dynamical and thermodynamic properties immediately upstream of the event are shown in Table 2. In particular, the presence or absence of favorable conditions for intense, localized and quasi-stationary convection is assessed.

**Table 2:** Meteorological fields averaged in the rectangular box 45°–45.5°N and 12.5°–13°E evaluated at 09 UTC of 26 September 2007, during the most intense part of the event.

	RTG	ROMS	AO	AOW	FLAT	SSTMOD	MFS
*U* (m/s)	10.3	12.2	11.7	11.6	10.5	11.4	12.0
*LFC* (m)	615	591	575	593	502	506	780
*N* (10^−2^ s^−1^)	1.4	1.1	1.1	1.1	1.1	1.2	1.2
*h* (m)	400	600	700	700	150	500	600
*h/LFC*	0.65	1.02	1.22	1.18	0.29	0.98	0.76
*C*_*s*_ (m/s)	9.0	11.0	12.0	12.0	8.0	10.2	11.0

First, the environmental wind speed *U* is smaller in the RTG run than in the other simulations. This is a direct consequence of the changes induced by the difference in SST, which, in the RTG run, is cooler on the northern and western Adriatic coast, and warmer along the Croatian coast. This modifies the circulation not only over the sea surface, but also near the coasts, in the areas most exposed to the incoming moist and warm flow from the Adriatic Sea, up to the Pre-Alpine ridge. There, the environmental flow in the RTG run is more westward-oriented than in the other simulations, producing a wider but shallower extension of cold air barrier flow extending from the Alps (not shown).

Second, in the RTG case the environmental profile is less favorable to the triggering of convection, due to the higher level of free convection (*LFC*) and to the greater Brunt-Väisälä frequency (*N*). In fact, while the latter indicates a higher resistance of the environment to vertical displacements, the former denotes a weaker tendency of the low-level parcels to reach *LFC* and trigger convection. This tendency is represented by the nondimensional parameter *h/LFC*^44,45^ where, following^46^, *h* does not represent the mountain height but the height of the cold pool, which behaves as an obstacle to the incoming southerly flow. In Table 2, *h* is evaluated as approximately equal to the height of the 1 °C potential temperature anomaly. The shallower vertical extension *h* of the cold pool and the higher *LFC* in the RTG experiment imply a value of *h/LFC* smaller than those observed in ROMS and in the coupled experiments, indicating weaker vertical displacement and tendency to convection. Figure 5 clearly shows that convection is much shallower and less intense in the RTG run compared to the coupled runs.

After this analysis, we go back to Fig. 4 and investigate more in details why the convergence line remains quasi-stationary in the coupled runs (Fig. 4c,d), while it changes significantly in the RTG experiment (Fig. 4a). This point is relevant considering that a stationary convergence line implies persistent convective triggering and rainfall in the same area. To identify if these conditions occur, here we follow the analysis performed by Miglietta and Rotunno^47^ and compare the theoretical cold pool propagation speed *C*_*s*_, associated with the cold air that has remained confined in the low-level (whose vertical extension is given by *h*), with the environmental wind *U*, evaluated in the rectangle located offshore (in latitude 45 °N–45.4 °N and longitude 12.5 °E–13.3 °E, based on Davolio et al.^15^), representative of the warm and moist air moving from the Adriatic toward the cold pool. We checked that ${C}_{s}$ and *U* have approximately opposite direction in all cases. Assuming that the cold-pool behaves as a density current, Benjamin^48^ and Klemp^49^ estimated the propagation speed as: ${C}_{s}= \sqrt{2g^{\prime}h}$, where ${g}{{^\prime}}=g ({\theta }_{1}-{\theta }_{0})/{\theta }_{0}$, being ${\theta }_{1}$ the potential temperature of the environment and ${\theta }_{0}$ that of the cold air mass. In RTG and ROMS runs, ${C}_{s}$ is smaller than the environmental wind *U* at 06 UTC (see Table 2) so that the cold pool penetrates inland. On the other hand, in the coupled simulations ${C}_{s}$ is higher than in RTG because of the higher depth *h*. The wind speed *U* also increases with respect to RTG, but not as much as ${C}_{s}$, so that the cold pool propagation is approximately counteracted by the environmental wind. The balance between these two flows represents the most favorable conditions for rainfall accumulation, as the uplift induced by convergence may remain stationary in the same position for several hours^47^.

## Conclusions

In coastal areas a detailed description of SST distribution plays a fundamental role for an accurate assessment of the MABL energy budget, with relevant implication for rainfall prediction^50^. However, despite new techniques of satellite acquisition, it is still difficult to get accurate SST data in coastal areas, in particular during extensive periods of cloud coverage.

This paper presents a numerical study using a high-resolution fully-coupled atmosphere–ocean–wave regional modelling system to analyze the sensitivity of an early Fall HPE in the surrounding of Venice. Compared to standalone atmospheric simulations using satellite data (run RTG), where the SST is available at coarse resolution every 6 h, and other SST datasets of different origin and resolution, the coupled systems AO and AOW provide significantly improved results in terms of precipitation amount, location, and timing of evolution. This is a consequence of the better representation of the fine-scale SST patterns due to the consistent treatment of air-sea interaction processes^12,28^. Therefore, the convergence of different air masses (cold, dry air from the Alpine regions and moist, warm air from the Adriatic basin), which is responsible for the generation of a back-building convective system, is better reproduced. Differences appear also comparing ROMS, where SST is updated at high-resolution, and AO/AOW cases, as only in the latter runs the heat fluxes at the MABL were consistently computed by the fully coupled system.

Also, as shown in Prtenjak^50^, 50% of summer days along the northern Adriatic coast are characterized by the presence of land-sea breeze systems. They usually develop between 08 and 18 UTC^50^, reaching their maximum intensity at around 15 UTC. In a semi-enclosed basin, such as the North Adriatic, during late summer the effect induced by breeze components may represent a secondary forcing for HPEs, which may locally add up to the main process, i.e. the large-scale dynamics. However, sea breeze may also contribute to the development of convection due to convergence with other breeze systems^51,52^, fronts, cold pools associated with previous precipitation, etc. The breeze dynamics are also influenced by the distribution of SST, which may locally affect the location of the coastal convergence line^53,54^. In this complex context, coupled atmosphere–ocean models, implementing high-resolution and frequently updated SSTs, represent more realistically coastal dynamics. Further studies are, however, needed to better assess their application to this phenomenology.

This study provides clear indications that the accurate simulation of localized rainfall events is eventually the result of a delicate interplay of energy balance that needs to be properly accounted for. A slight adjustment of the wind field, which may be provided by the different heat distribution along the coast generated by an ocean circulation model, can indeed change significantly the amount and location of forecast rainfall. In the same way, stormy conditions may enhance the role of surface waves in affecting momentum across the MABL^28^. Although limited to one case study, results presented here have a general value, on the one hand about the relevance of the use of model coupling and, on the other hand, about the limitations of operational NWP approaches when constant or coarse resolution SSTs are used as surface boundary conditions. The Mediterranean region is frequently affected by HPEs, often related to the presence of convergence lines^55,56^, which are associated to the clashing of cold and dry air versus highly-unstable, warm and moist masses. In our case study, the use of high resolution and frequently updated SST improved the timing performance of the convergence line (well depicted by ROMS, AO and AOW runs), thus allowing a better simulation of the rainfall amount and location. Having said this, in order to provide improved HPE and hazard predictions in other coastal regions via coupled numerical tools, there is still a strong need to explore a broad range of conditions, as well as to carry out more extensive sensitivity studies.

## Supplementary information

Supplementary Figures.

## Data Availability

The datasets generated and/or analysed during the current study are available from the corresponding author on reasonable request.
