# Author Correction: Simulation of a flash-flood event over the Adriatic Sea with a high-resolution atmosphere–ocean–wave coupled system

**DOI:** 10.1038/s41598-025-00864-z

**Published:** 2025-05-23

**Authors:** Antonio Ricchi, Davide Bonaldo, Guido Cioni, Sandro Carniel, Mario Marcello Miglietta

**Affiliations:** 1 Department of Physical and Chemical Sciences, University of L’Aquila, Via Vetoio (Coppito 1, Edifcio “Renato Ricamo”), 67100 L’Aquila, Italy; 2 Center of Excellence in Telesensing of Environment and Model Prediction of Severe Events (CETEMPS), Via Vetoio (Coppito 1, Edifcio “Renato Ricamo”), 67100 L’Aquila, Italy; 3 CNR-ISMAR, Institute of Marine Sciences, Arsenale Tesa 104, Castello 2737, 30122 Venice, Italy; 4 Max Planck Institute for Meteorology, Hamburg, Germany; 5 STO CMRE, V.le San Bartolomeo 400, I-19126 La Spezia, Italy; 6 CNR-ISAC, Padua/Lecce, Italy

Correction to: *Scientific Reports* 10.1038/s41598-021-88476-1, published online 30 April 2021

The original version of this Article contained an error in Affiliation 3, which was incorrectly given as ‘CNR-ISP, Institute of Polar Sciences, via Torino 155, I-30172, Venezia Mestre, Venice, Italy.’.

The correct affiliation is listed below:

CNR-ISMAR, Institute of Marine Sciences, Arsenale Tesa 104, Castello 2737, 30,122, Venice, Italy

