# Supplementary material for: Simulation of a flash-flood event over the Adriatic Sea with a high-resolution atmosphere–ocean–wave coupled system
Source: Sci Rep. 2021 Apr 30;11:9388. doi: 10.1038/s41598-021-88476-1 (PMC8087830; doi:10.1038/s41598-021-88476-1)
Supplement: Supplementary file 1 — Supplementary Figures. [file 41598_2021_88476_MOESM1_ESM.pdf]

# Simulation of a flash-flood event over the Adriatic Sea with a high-resolution atmosphere-ocean-wave coupled system

Antonio Ricchi[1,2,3], Davide Bonaldo[3], Guido Cioni[6], and Sandro Carniel[3,5], Mario Marcello Miglietta[4]

[1]University of L'Aquila, Aquila, Italy, [2]CETEMPS, Rome, Italy
[3]CNR-ISMAR, Arsenale, Tesa 104, Castello 2737/F, 30122 Venice, Italy, [4]CNR-ISAC, Padua/Lecce, Italy, [5]NATO, La Spezia, [6]Max Planck Institute for Meteorology Hamburg, Germany

Correspondence to Antonio Ricchi email : antonio.ricchi@univaq.it

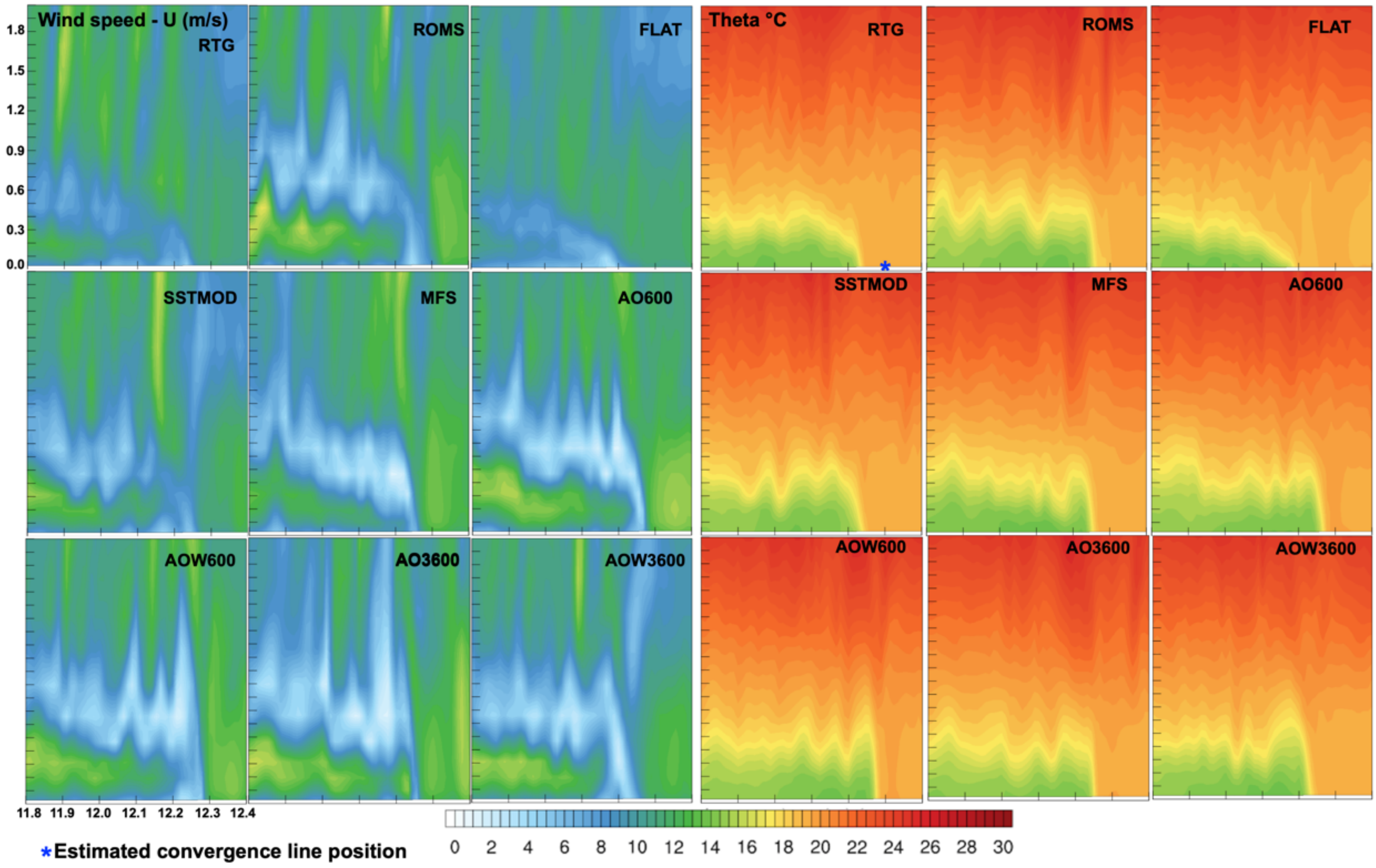


**Supplementary Figure 1:** Cross section of the wind component (m $s^{-1}$) and of Theta-e (°C) along the convergence line between the ground and the altitude of 2000 m.

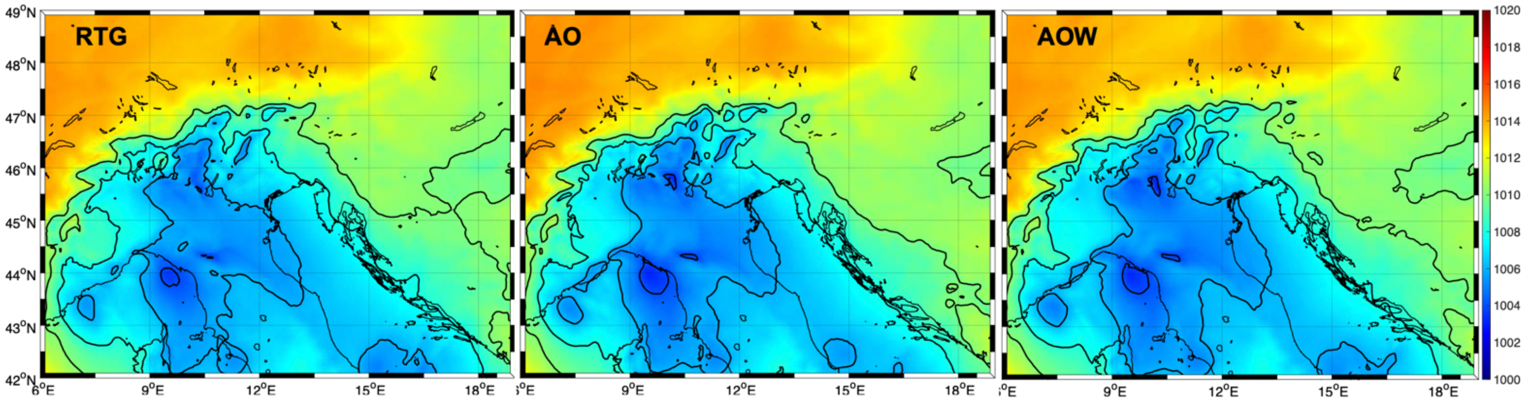


**Supplementary Figure 2**: Mean sea level pressure fields (hPa) for the RTG, AO and AOW simulations.

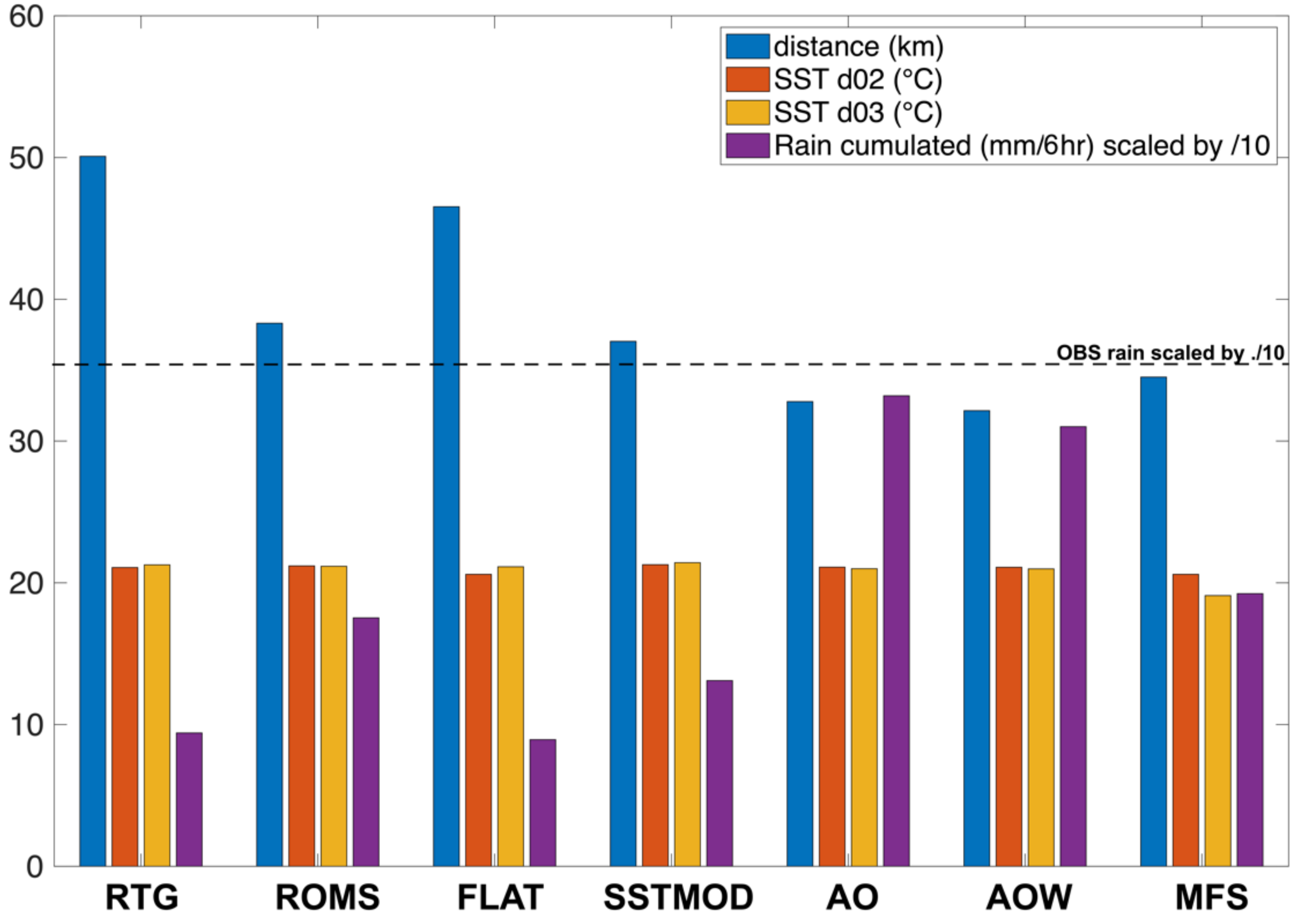


**Supplementary Figure 3**: Histograms, for all simulations, of the average distance of the simulated storm cell from the observed one; the mean SST in the D02 and D03 domain and the cumulative precipitation from the different simulations (scaled by a factor 10)
